# Combined metallomics and metabolomics reveal impact of metal homeostasis on biological pathways in *C. elegans*

**DOI:** 10.1007/s00216-025-06306-z

**Published:** 2026-01-09

**Authors:** Bastian Blume, Philippe Schmitt-Kopplin, Bernhard Michalke

**Affiliations:** 1 Research Unit Analytical BioGeoChemistry, Helmholtz Center Munich - German Research Center for Environmental Health (GmbH), 85764 Neuherberg, Germany; 2 Chair of Analytical Food Chemistry, TUM School of Life Science, Technical University of Munich, 85354 Freising-Weihenstephan, Germany

**Keywords:** Metal speciation, *C. elegans*, Metal treatment, Parkinson’s disease, FT-ICR-MS, Metabolomics

## Abstract

Trace metals and metal homeostasis play an essential role in cell metabolism, and an imbalance in this careful balance leads to pathological changes. In neurodegenerative diseases such as Parkinson’s disease, trace metals and metal species are increasingly recognized as key factors in disease progression in mechanisms such as ferroptosis. In this study, we combined modern analytical techniques like Fourier transform ion cyclotron resonance mass spectrometry (FT-ICR-MS) with metal-free high-pressure liquid chromatography inductively coupled plasma mass spectrometry (HPLC-ICP-MS) to analyze metabolomic and metallomic changes in the model organism *Caenorhabditis elegans* under conditions of induced metal homeostasis imbalance. Our results identified zinc as a key player in the regulation of free metal species of iron and manganese. Furthermore, iron exposure resulted in distinct metabolomic patterns indicative of impaired energy metabolism, suggesting an exhaustion of cellular metabolic energy capacity.

**Graphical abstract:**

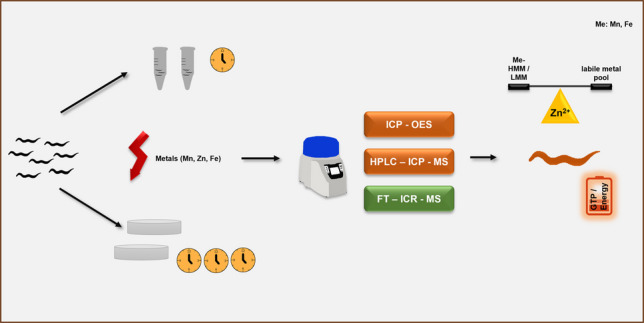

**Supplementary Information:**

The online version contains supplementary material available at 10.1007/s00216-025-06306-z.

## Introduction

Metals such as iron, manganese, and zinc are essential for biological organisms, being involved in cellular metabolism as active centers or co-factors of enzymes in all six enzyme classes, transferring oxygen, acting as intracellular secondary messengers, and modulating synaptic transmission [1]. Iron is an important mediator of the respiratory system as an electron donor and acceptor and therefore indispensable for cellular energy production and is part of enzymes catalyzing redox reactions. Inorganic iron is potentially harmful to all cell components, due to its redox activity. Reactive oxygen species are produced by iron in a reaction named the Haber-Weiss reaction [2]. Therefore, the level of inorganic iron is at a relatively low level and well controlled. With an electron configuration of manganese of [Ar] 3d5 4s2, the most stable ion in aqueous solution is Mn^2+^, but oxidation states from +II to +VII are possible. Therefore, manganese is considered redox active, even though less active compared to iron with its common oxidation states of +II and +III. Manganese has an important function as a co-factor for various enzymes. Manganese complexes derive the active center of enzymes for redox reactions. Mn-superoxide dismutase (Mn-SOD) is one of the most important examples. In mitochondria, manganese has its function in neutralizing reactive oxygen species, which are constantly generated in the respiratory chain reaction [3]. More than 300 specific enzymes have structural domains containing cysteine and histidine residues for the complexation of zinc [4, 5]. Zinc induces conformational changes activating the enzymes. Conclusively, the removal of zinc goes along with the loss of functionality. However, the process is reversible by reconstitution. Therefore, zinc competes with other free metal ions with similar ionic radii and coordination. Mis-metalation can occur whenever the level of metal pools is disturbed, leading to misfunction of proteins [6, 7]. Unlike manganese and iron, zinc has a filled “*d*” orbital and therefore is not a redox-active metal. Intracellular metal species levels are in a physiological state in a tightly balanced equilibrium between high-molecular-mass (HMM, > 100 kDa), low-molecular-mass (LMM, <100 kDa), and inorganic species. To sustain the total metal content constantly, cells can regulate the expression of genes responsible for metal uptake, such as the divalent metal transporter. The activation or deactivation follows a complex process, which starts with metal-regulating factors sensing the metal concentration and influencing the transcription, alternative splicing, translation, mRNA stability, protein activity, and protein stability. The mechanisms are not fully understood, but current achievements are reviewed by Bird [8]. The inorganic iron level is regulated by binding and storing iron in ferritin. However, the exact binding and releasing processes are not fully understood [9]. Viral infections [10, 11], environmental factors [12], genetic defects [13], and aging [14] affect the regulation mechanisms of metals and therefore alter the metallome. Similar risk factors are known to cause Parkinson’s disease progression. Different findings indicate an imbalance of metal homeostasis or a dysfunction of regulatory mechanisms [15], resulting in ROS generation and neurodegeneration [16, 17]. Although the development of Parkinson’s disease has been the focus of scientific research for decades, no definitive biochemical understanding of the underlying mechanisms has yet been gained. However, metals seem to play a crucial role in disease progression, as recently reviewed by Chen et al. [18]. In our study, we focused on the alteration of the metallome when metal homeostasis is out of balance in the model organism *C. elegans*. We induced the imbalance by acute and chronic metal exposure and determined the distribution of LMM, HMM, and inorganic metal species after extraction, using modern hyphenation ICP-MS techniques, as well as metabolomic approaches using Fourier transform ion cyclotron resonance mass spectrometry. As model organisms, we used the nematode *Caenorhabditis elegans* (*C. elegans*) with its fully developed nervous system. *C. elegans* meets the requirements for this fundamental study on the metallome and metabolome with its short reproducing cycles, its fully developed nervous system, well-conserved basic cellular mechanisms for metal regulation, and many proteins with human homologues [19]. Non-apoptotic, regulated forms of cell death, such as ferroptosis, have been reasoned to be the mechanism of cell death after induced iron imbalance in *C. elegans* in numerous studies [20–22]. However, direct translation to more complex systems or human diseases is not possible. The results obtained in this study may shed light on previously unknown metal regulation mechanisms in *C. elegans*, from which possible explanations for disease progression, such as PD, can be inferred. Nevertheless, these inferences are theoretical and will require further validation in humans or more complex organisms than *C. elegans*. In the following experiments, metal concentrations considerably higher than in physiological or environmentally relevant levels are applied to induce an imbalance in metal homeostasis, without instantly killing the organism. Concentrations chosen were elaborated in prior work to induce an imbalance, but being still sustainable by the organism [23].

## Material and methods

### Material

Sodium chloride (NaCl, analytical grade) was ordered from Fluka® Analytical (Munich, Germany). Agar granulates for NGM-plates were ordered from BD Diagnostics (Franklin Lakes, USA). Methanol (MeOH, LC-MS grade), acetic acid (≥ 99.99%), HCl Suprapur® (30%), FeSO_4_ × 7 H_2_O, MnSO_4_ × H_2_O, and ZnSO_4_ × 7 H_2_O were ordered from Sigma-Aldrich (Darmstadt, Germany). Synthetical quartz beads (diameter: 0.315–0.5 mm) for homogenization were purchased from Gaßner Glastechnik GmbH (Oberhaching, Germany). HNO_3_, H_2_O_2_, and TRIS (≥ 99.99%) were ordered from Carl Roth GmbH and Co. KG (Karlsruhe, Germany). HNO_3_ was purified by sub-boiling distillation before use.

### Cultivation and treatment of *C. elegans*

Wild-type N2 (Bristol, UK) strain of *C. elegans* was used for all experiments. It was maintained at 20 °C following the protocol of Brenner [24]. *Escherichia coli* (*E. coli*) *OP50*, used as a food source for the nematode, was cultivated in Luria-Bertani medium and seeded on nematode growth medium-plates (NGM-plates). To achieve age synchronization, the culture was bleached on the 4th day after hatching with a basic hypochlorite solution [25]. The eggs were hatched in M9-buffer (22 mM KH_2_PO_4_, 22 mM Na_2_HPO_4_, 85 mM NaCl, and 1 mM MgSO_4_) for 13 h, fed with *E. coli* for 2 h, washed again with M9-buffer, followed by 2 × washing with 86 mM NaCl solution. The L1 were counted and suspended to a final concentration of 200 k L1 per mL in 86 mM NaCl for immediate use.

#### Acute exposure

500 µL worm suspension was added to a 2 mL vial, filled up with 86 mM NaCl and a predefined amount of metal stock solutions to reach the final concentrations of 5 mM, 50 mM, and 50 mM for iron, manganese, and zinc treatments, respectively. The concentrations have been elaborated prior in life span experiments and have been applied already in prior published work [23] to induce an imbalance in the metal homeostasis. Each concentration and the control were carried out in six replicates for metallomic studies and ten replicates for metabolomic studies. Capped vials were incubated for 30 min at 20 °C and 1000 rpm. Subsequently, the samples were centrifuged at 10,000 × g and 4 °C for 5 min. The supernatant was aspirated, and the pellet was washed two times with 86 mM NaCl and three times with MiliQ® water. The samples were stored until further use at −80 °C.

#### Chronic exposure

Concentrated *E. coli* (suspension of 1 mL *E. coli* pellet in 4 mL LB-medium) was provided with a defined amount of metal stock solution to reach a final concentration of 1 mM, 10 mM, and 10 mM for iron, manganese, and zinc treatments, respectively. The concentrations have been elaborated in experiments to achieve non-lethal conditions but a significant metal uptake to induce an imbalance in this specific metal concentration. The metal uptake at different treatment concentrations has been elaborated and has been published in a previous study [23]. The mixtures were incubated under rigorous shaking for 1 h. 1 mL *E. coli* or *E. coli-*metal mixture was seeded on 90 mm NGM-plates and dried for 3 h. Each experiment was carried out in six replicates for metallomic studies and ten replicates for metabolomic studies. 10,000 freshly hatched L1 were transferred to the plates and incubated for 3 days at 20 °C. When the *E. coli* was almost depleted, before the egg laying started, the worms were collected from the plates using 86 mM NaCl, washed two times with 86 mM NaCl, and three times with MilliQ® water. The samples were stored until further use at −80 °C.

### Extraction and digestion procedures

#### Metallomic studies

The extraction of metal species was carried out using a previously developed protocol [26] and optimizing the extraction medium for sustaining metalloprotein species based on a previously published protocol [27]. The extraction medium was 10 mM Tris in MilliQ®. The pH of 7.4 was adjusted with HCl and NaOH. The aqueous phase was aspirated and collected. The pellet and aqueous phase were stored at −80 °C until measurement. The quartz beads and the worm pellet were transferred to 10 mL quartz vials, and the ashing-mixture (500 µL MilliQ® water, 250 µL H2O2 (30%), and 250 µL HNO3) was added. Vials were capped and placed in a digestion apparatus for 6 × 100 mg samples and PTFE digestion vessels with quartz glass tubes (12 h, 180 °C). After digestion, the samples were diluted with 10 mL MilliQ®, separated from the quartz beads, and the metal content was determined with ICP-OES.

#### Metabolomic studies

The extraction of metabolites was carried out with the homogenization protocol as described before [26] using 70% MeOH as the extraction medium, which was used for metabolomic studies in *C. elegans *[28]. Samples were collected, washed 5 times with MilliQ-water, transferred to tubes containing quartz beads (200 mg per sample), overlaid with a total volume of 400 μL methanol. Homogenization was carried out at −10 °C at 6000 rpm with 3 × 20 s homogenization intervals and a 30 s pause, using a Precellys bead tube homogenizer. Samples are normalized to the number of worms. The supernatant was collected, the remaining pellet was washed two times with MilliQ® water, digested as described before, and used for elemental determination with ICP-OES. The metabolite extract was diluted 1:10 for acute treatment and 1:50 for chronic exposure with 70% MeOH.

### Total metal determination with ICP-OES

100 µL extract were filled up to 3 mL with 2% sub-boiled HNO_3_, and 5 mL of digestion media separated from quartz beads were used for total metal determination with ICP-OES (SPECTRO ARCOS, Amtek, Inc., Berwyn, USA). Six biological replicates and two technical replicates were determined. The quantification of metals was based on external calibration with certified standards, with a linearity better than 0.99998 for each metal. Periodically, after 10 measurements, blank and quality control (QC) measurements were carried out. The certified reference material BCR185R (bovine liver) was measured frequently for quality assurance. Elemental lines were checked daily with standard solutions for shifts or weak performance. The following elemental emission lines were chosen for the quantification (in nm): Fe—59.939, Mn—257.610 and Zn—313.857.

### Iron, manganese, zinc speciation

The extracts were thawed and diluted 1:1 with 10 mM Tris (pH: 7.4) before measurement. The separation was performed on the metal-free NexSAR 200 (Perkin Elmer, Toronto, Canada) equipped with the YMC-Pack Diol-300 (300 × 8 mm, 5 µm, separation range 2–700 kDa) (YMC, Kyoto, Japan) and a PEEK column packed with TOYOPEARL® HW-40S (250 × 8 mm, separation range 100–2000 Da) (TOSOH, Tokyo, Japan). An isocratic method [29] was modified to a stepwise isocratic method to achieve higher performance for manganese speciation (Table 1). Eluent A was 50 mM ammonium acetate (pH = 5.6) and Eluent B was 500 mM ammonium acetate and 5% MeOH (pH = 8) [29]. The detection was carried out with the ICP-MS system NexION 300 d (Perkin Elmer, Toronto, Canada). The system was used in dynamic reaction cell (DRC) mode. The reaction gas was ammonia with a flow of 0.6 mL/min. The plasma gas flow was set to 16 L Ar/min, the nebulizer gas was set to 0.96 Ar/min, the dwell time was set to 100 ms, and the RF power was set to 1250 W. Parameters, such as gas flow (plasma, auxiliary, collision cell, carrier) and lenses, were optimized daily for low oxide ratio of < 1% (^140^Ce^2+^/^140^Ce^+^) with background counts of < 0.1 cps and maximum sensitivity. The Syngistix (Perkin Elmer, Toronto, Canada) and Clarity™ Software (Data Apex, Prague, The Czech Republic) were used for operating the instruments. Ferritin, γ-globulin, apo-transferrin, human serum albumin, β-lacto globulin were used for the mass calibration. A regression of the reciprocal retention time against mass in kDa resulted in a calibration curve: *y*(*x*) = −7.87E−08x^2^ + 1.24E−04x + 3.9E−02 for chronic exposure and *y*(*x*) = −2.4E−07x^2^ + 2.1E−04x + 3.5E−02 for acute exposure with *R*^2^ of 0.9998 and 0.9998, respectively.

**Table 1:** Conditions for the adjusted stepwise isocratic gradient LC-method

Step	Time	A	B	Flow
	[min]	[%]	[%]	[mL/min]
1	0	90	10	0.75
2	40	90	10	0.75
3	45	10	90	0.75
4	85	90	10	0.75
5	100	90	10	0.75

### Data treatment and statistics

#### Metallomics

Obtained spectra were exported from Clarity™ as.txt files for each isotope. The.txt files were automatically reprocessed in R-studio and exported in.txt files for further processing. Each spectrum was loaded in PeakFit®. Baseline subtraction, peak annotation, and peak fitting were carried out, achieving *R*^2^ > 0.95. Numeric data was exported as.txt. Further processing, statistical analysis, and generating graphs were carried out in R-studio. Packages used in R were as follows: ggplot2, dplyr, ggpubr, openxlsx, patchwork. Comparisons between control and treated samples were carried out with Student’s *t*-test, with *p*-values < 0.05 as significant (*), < 0.01 as moderately significant (**), and < 0.001 as highly significant (***). Error bars in the bar charts refer to the standard error of the mean (SEM).

#### Metabolomics

Metabolite profiling was carried out on a Bruker solariX ion cyclotron resonance Fourier transformation mass spectrometer (Bruker Daltonics GmbH, Bremen, Germany) equipped with a 12 Tesla superconducting magnet (Magnex Scientific Inc., Yarton, GB) and an APOLO II ESI source (Bruker Daltonics GmbH, Bremen, Germany). Obtained mass spectra in negative ionization mode were recalibrated internally by linear mass calibration up to 800 m/z using an in-house calibration list of about 150 recurrent compounds present in all samples. A maximal mass error below 0.1 ppm was tolerated. The data was exported as a list of mass and intensities at a signal-to-noise ratio of 3 using Bruker Compass Data Analysis 5.0 (Bremen, Germany). The data was then reprocessed using an in-house R-based (Version 4.2.2) package. Peaks were aligned within a threshold of 0.5 ppm for spectra obtained with negative ionization. Satellite peaks and isotopes were removed, and a mass defect filter was applied (exact parameters are provided in the supplementary information). Annotations were made based on a mass different network algorithm based on a biological network using in-house software and a list of known metabolites (NetCalc) [30]. Annotations with NA content over all samples with > 0.75 were removed prior to statistical analysis. Annotations were matched against the HMDB database and an in-house list of metabolites known to be involved in major biological pathways. Different treatment groups were then compared to the control using the two tailed Student’s *t*-test for each of these metabolites (*n* = 55). Because multiple metabolites were tested, the resulting *p*-values were corrected for multiple comparisons using the Benjamin-Hochberg (BH) method. Metabolites with BH-adjusted *p*-values (*q*-values) < 0.05 were considered significant.

## Results and discussion

### Increased metal levels after treatment

The metal content has been determined by measuring the content of the extract and the digested pellet with ICP-OES and normalization on the number of worms. Acute (Table 1) and chronic (Table 2 and Table 3) treatments reveal elevated metal levels for exposed worms regarding their corresponding metal. Additionally, manganese and iron exposure decrease the total level of zinc in acute treatment (*p*-values: 0.008 and 0.021). This might be explained by an activation of zinc excretion due to increased iron or manganese level. Chronic exposure to zinc decreases the level of manganese significantly (*p*-value = 0.001). Furthermore, the zinc level is decreased under chronic exposure with manganese (*p*-value = 0.03). This finding indicates a cross-metal effect and an activation of excretory mechanisms or inhibition of absorption pathways [31] of zinc and manganese. However, iron levels are not affected by zinc or manganese exposure.

**Table 2:** Metal contents per worm measured from the extract and digested pellet of acute iron, manganese, zinc treated, as well as control. Measurements were carried out with ICP-OES. Significance levels of differences between control and treated groups are presented with *, **, and *** depending on the levels of significance (*p*-value: < 0.05, < 0.005, and < 0.0005)

Treatment	Fe 259.941	Mn 257.611	Zn 213.856
	pg/worm	pg/worm	pg/worm
Control	13.83 ± 0.58	1.7 ± 0.06	134.7 ± 15.35
Iron	354.6 ± 45.5***	2.02 ± 0.25	86.31 ± 36.4*
Manganese	15.62 ± 1.33*	66.21 ± 8.82***	57.28 ± 49.81*
Zinc	13.88 ± 0.71	1.87 ± 0.06	218.8 ± 13.84***

**Table 3:** Metal contents per worm measured from the extract and digested pellet of chronical iron, manganese, zinc treated, as well as control. Measurements were carried out with ICP-OES. Significance levels of differences between control and treated groups are presented with *, **, and *** depending on the levels of significance (*p*-value: < 0.05, < 0.005, and < 0.0005)

Treatment	Fe 259.941	Mn 257.611	Zn 213.856
	pg/worm	pg/worm	pg/worm
Control	85.11 ± 6.59	27.22 ± 3.44	407.11 ± 198.41
Iron	551.12 ± 37.58***	26.03 ± 0.89	268.42 ± 76.9
Manganese	75.1 ± 13.57	1219.04 ± 24.32***	181.18 ± 40.61*
Zinc	122.13 ± 39.91	15.05 ± 2.67***	979.53 ± 120.44***

### Effect of metal exposure on the distribution of metal species

**Fig. 1:**
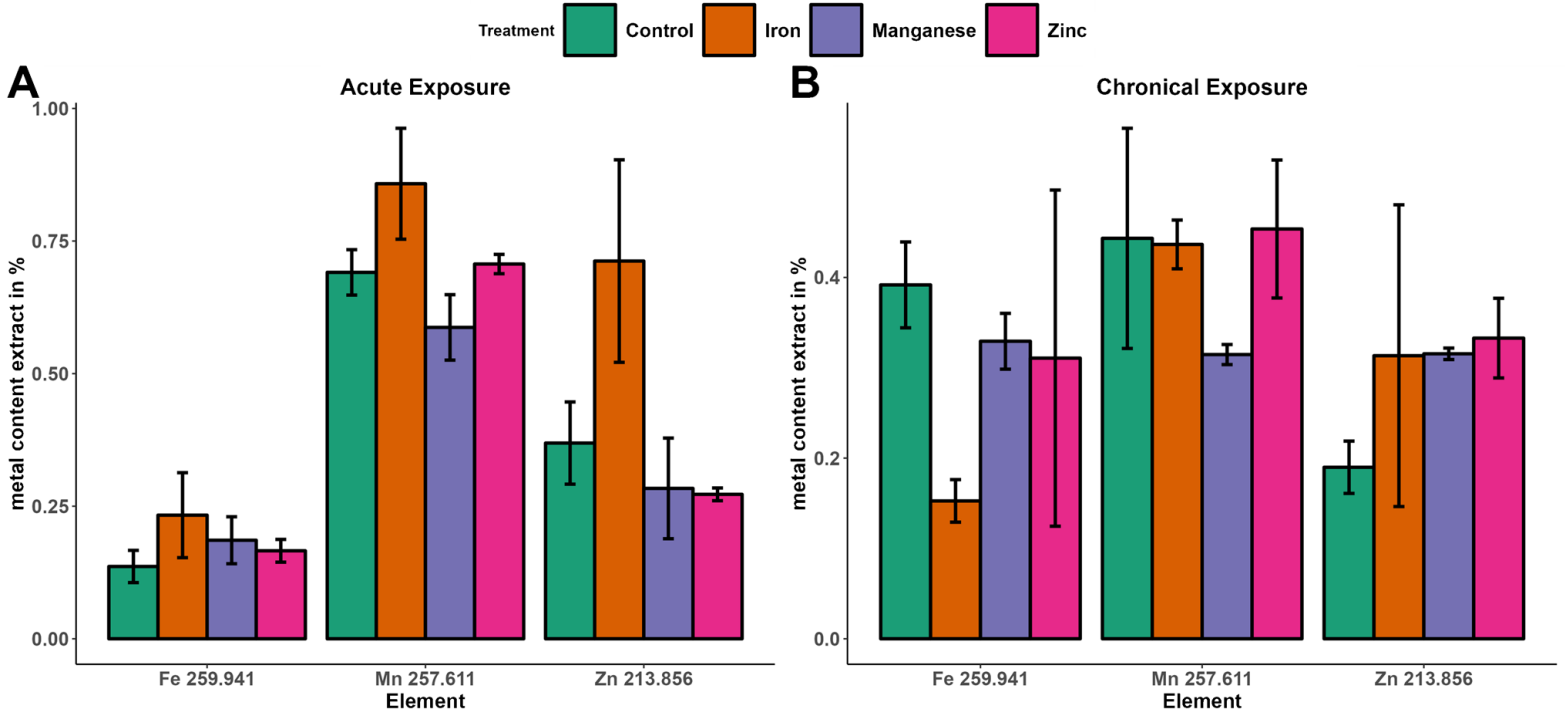
Distribution of total metal content between the extract and the pellet. Presented is the percentage of total metal content in the extract of L1 after acute treatment of manganese (purple), iron (orange), zinc (pink), and control (green) (**A**) and YA after chronic treatment of manganese (purple), iron (orange), zinc (pink), and control (green) (**B**)

Absorbed metal is first added to the labile metal pool and distributed to the organism or cell organelles. Metals can be loosely bound in the LMP, bound to low-molecular-mass or to high-molecular-mass molecules such as ferritin. Metal species occur in the cytosol, the cell organelles, or are bound to membrane proteins. Redox-active metals in their free or loosely bound forms are potentially causing ROS-induced cellular damage. Conclusively, mechanisms to bind and store free metals are well developed and highly conserved in living organisms. *C. elegans* does not preserve mechanisms for long-term storage of manganese and zinc, as being reported from sophisticated organisms. In *Homo sapiens*, manganese is long-term stored in bone matter, with about 40% of the body’s total metal content [32], and zinc is mainly stored in skeletal muscle with about 60% and bones with 30% of the body’s total metal content [33]. However, basic mechanisms to sustain the cellular metal concentration exist in *C. elegans*, which key components we extracted. The aqueous extract of *C. elegans* samples contains soluble metalloproteins and inorganic metal species, whereas insoluble and membrane proteins, undigested cell organelles, and cell fragments remain in the pellet fraction. Comparing treated samples with controls revealed shifts towards elevated iron, manganese, and zinc in the extract under acute exposure to iron (Fig. 1A). These findings can be explained by insoluble or membrane proteins capable of reversibly binding iron, manganese, or zinc, whose binding spots are occupied by treated iron. One interesting example described before in the context of metal binding ability is the myosin family. The class contains a various amount of different insoluble proteins consisting of divalent metal binding sites, capable of binding manganese, magnesium, and calcium ions [34, 35]. In chronic treatment, the relative level of manganese and iron in the extract decreases (Fig. 1B). This can be seen as an organismal response to the excessive intake of metals and the attempt to keep the metal equilibrium between bound and unbound metal in balance. Further, we studied the metal species in the extract and discussed the results in the following paragraph.

We investigated the distribution of ingested metal between different metal species such as high-molecular-mass molecules (HMM > 100 kDa), low-molecular-mass molecules (LMM < 100 kDa), and inorganic species (inorg < 0.5 kDa) after treatment with Fe, Mn, and Zn, compared to a control group. The suitable analytical technique used was size exclusion chromatography hyphenated with ICP-MS (SEC-ICP-MS). Important examples of metalloproteins or complexes are summarized below (Table 4). Inorganic metal species are located inside cells in the cytosol or organelles such as the Golgi apparatus, lysosomes, or mitochondria. Furthermore, we assumed cross-metal effects between the metal species and therefore investigated Zn, Fe, and Mn species for all three treatment experiments, respectively.

**Table 4:** Fundamental metalloproteins and metal species with our corresponding classification in HMM, LMM, and inorganic species

Species	Peptides/complexes	Molecular weight [kDa]	Metal containing	Ref
**HMM**	Ferritin	400–500	Fe	[36, 37]
	Transferrin receptor	-	Fe, Mn	
	Astacin	592.8	Zn	
	Hemosiderin	-	Fe	
	Glutamine synthetase	500–750 (aggregate)	Mn, Mg	
	Pyruvate carboxylase	-	Mn	
**LMM**	Superoxide dismutase	32.5	Cu, Zn, Mn	[38–41]
	Mn-dependent phosphatases	78	Mn	
	Transferrin	76	Fe, Mn, Zn	
	Cytochrome p450	45–65	Fe	
	Metallothionein	0.5–14	Mn, Zn	
	Carbonic anhydrase	30	Zn	
	Ferritin family of iron binding proteins (FET-3)	20–45	Fe	
	Zinc transporter (ZIP-1, 2, 4, 7)	20	Zn	
	Arginase	35–38	Mn	
**Inorganic**	Low-molecular weight complexes	< 1	Zn, Fe, Mn	[42]
	Liposomes	-		

**Fig. 2:**
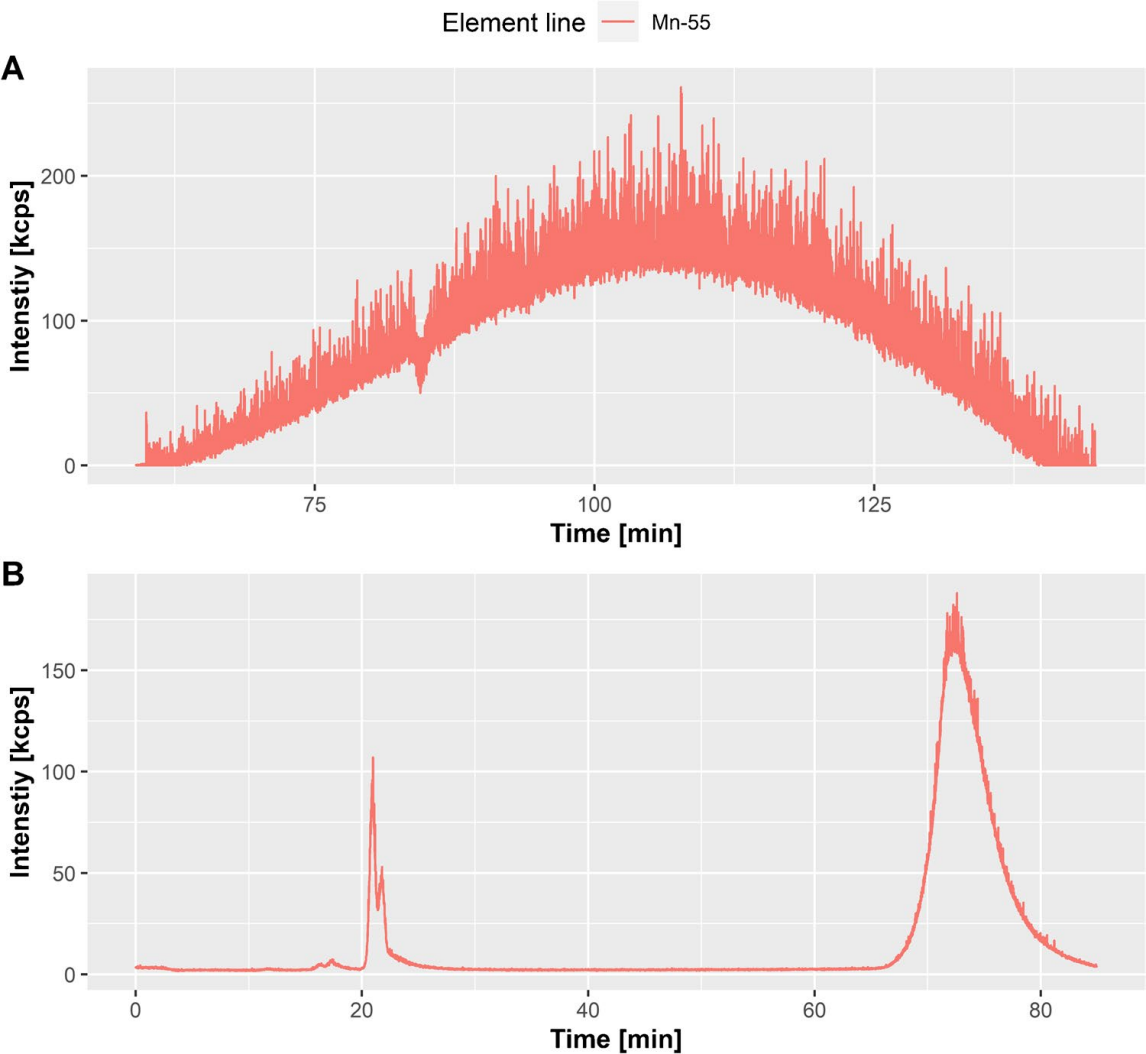
Chromatogram of manganese species from 60 to 140 min using the isocratic method (**A**). Chromatogram of manganese species using the modified stepwise isocratic method, optimized for manganese species with high column interactions (**B**)

In the case of manganese, we noticed a very broad manganese peak from 70 to 140 min when using an isocratic gradient (Fig. 2A). Elution after 60 min states strong interactions with the column material, which indicates the hydrophobic character of the species. Therefore, we modified the separation method to obtain better performance, resulting in a stepwise isocratic method with increased salt and organic concentration. A sharp manganese peak at 70 min (Fig. 2B), which we attributed to the release of manganese from a hydrophobic manganese species, was achieved, while species eluting before were identical. Further investigations revealed the strong attachment of the hydrophobic manganese species to the column material. Interestingly, the attachment to the column material does not neglect the binding affinity of manganese, as subsequent measured manganese from free metal mix standard solutions (100 ppb, Mn_(aq)_, Fe_(aq)_, Zn_(aq)_) was also bound. Backflushing the column for several minutes resulted in an elution and regeneration of the column material for free manganese. We would like to mention that these findings may be interesting in future work in this field. The characterization of this species is not the subject of this publication. However, by being aware of its existence and by the optimization of the elution conditions, we were also able to measure the amount of manganese released. Future investigation for the precise character of the fraction collected while backflushing is needed and promising, as they can contribute to the understanding of how manganese is bound inside of the Golgi apparatus, lysosomes, or mitochondria as manganese-rich cell organelles. Previous investigations showed manganese enrichment in the Golgi apparatus under excessive manganese exposure [43], which might be the origin of this species. At this part of our research, we cannot be certain, but we suggest this predominant manganese species to be located within the Golgi apparatus bound to hydrophobic membrane proteins.

#### Acute treatment

**Fig. 3:**
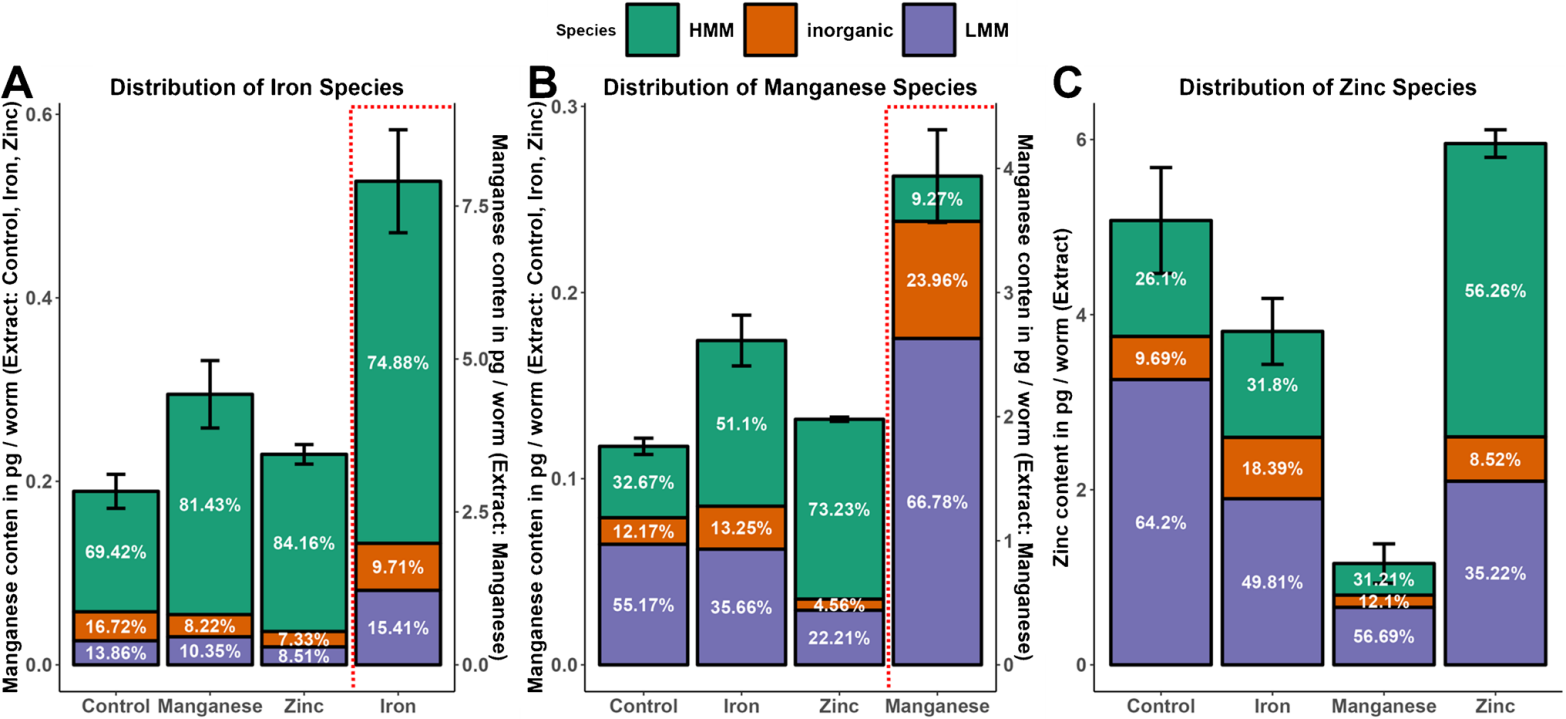
Distribution of high-molecular-mass (HMM), low-molecular-mass (LMM), and inorganic Fe (**A**), Mn (**B**), and Zn (**C**) species after acute Fe, Mn, Zn treatment, and control (*x*-axis). Determination of HMM, LMM, and inorganic species was carried out with SEC-ICP-MS and presented in % and against the total metal content normalized on the number of worms (*y*-axis) determined in the extract with ICP-OES

The distribution of iron species after metal exposure does not differ significantly from the control. Organisms from *C. elegans* to *Homo sapiens* consist of very well-developed mechanisms for maintaining the balance between bound and unbound iron, since free iron is redox active and induces oxidative stress. Therefore, iron in L1 is mainly bound to HMMs, with minor parts in the inorganic and LMM fractions. Exposure to iron significantly increases the total iron level in the extract (*p*-value: 0.000008). In absolute terms, the inorganic iron is higher. But the relative amount of inorganic HMM-bound iron is lower compared to the control. This fact is explained by the high ability of ferritin to bind and store iron until its capacity is exceeded. Remarkably, LMMs bind iron and support maintaining the balance between bound and unbound iron. An initial assumption is that the LMM protein binds iron and stabilizes the level of unbound iron. One possible family of proteins explaining this finding may be the metallothionein (MT) family. MTs have been shown earlier to bind iron in situ [44]. Manganese and zinc treatment lowers the proportions of LMMs and inorganic iron, while iron is transferred to the HMMs (Fig. 3A). Manganese is essential for organisms in the active center of enzymes or as a cofactor for the regulation of enzymes. Superoxide dismutase (SOD), arginase, Mn-dependent phosphatases, glutamine synthases, and pyruvate carboxylase, often localized in mitochondria, contain manganese as an active center. In L1, manganese is mainly bound to LMMs and HMMs, which is not surprising in this early stage of development. Interestingly, the distribution of manganese shifts from inorganic and LMMs towards HMM in the case of zinc exposure. This indicates that zinc and manganese compete for LMM binding sites. However, the exact mechanisms of how manganese is transferred from LMMs to HMMs species under zinc exposure are unclear. Manganese exposure elevates the level of manganese in the extract significantly (*p*-value: 0.000003). A large amount is bound to LMMs species but also remains in the inorganic fraction. HMMs seem to bind only minor parts of manganese in the case of massive manganese influx. Manganese is discussed to be stored in the nucleus, Golgi apparatus, lysosomes, or mitochondrion, where high concentrations of manganese have been observed [45]. However, not much is known about how manganese is bound and stored in these organelles. Our results indicate that manganese might be ionic or bound loosely, while being released when cell organelles are digested in L1. Ferritin-like storage proteins are not known for manganese in *C. elegans* as well as *Homo sapiens *[46]. Zinc is distributed mainly to the LMM fraction. Treatment with iron decreases the zinc level in the extract, as well as the distribution of zinc in the LMM fraction, while increasing the percentage of zinc in the HMM and inorganic fractions. Together with the observed decrease of iron in LMMs after zinc treatment, we conclude iron competing with zinc bound to LMMs, which results in the release of zinc to the inorganic fraction. Furthermore, ferric iron may oxidize the cysteine residues of MTs, which results in the release of zinc in a mechanism suggested by Maret et al. and further investigated in situ with dithiopyridine as an oxidative agent [47]. Interestingly, the level of zinc in the extract of manganese exposed worms is significantly decreased, while the distribution to HMM, LMM, and inorganic fractions remains (Fig. 3C). Referring to our prior findings of decreased total zinc levels after acute manganese and iron exposure, we suspect first the release of inorganic zinc from the LMM, HMM, and insoluble fraction followed by zinc-excretion [48]. This indicates an excretion mechanism for zinc, controlled via inorganic zinc levels, which in the case of manganese exposure results in zinc deficiency. Zinc regulates several cellular processes as part of zinc-finger proteins (ZNFs), being able to interact with DNA, RNA, poly-ADP-ribose, and various other proteins [49]. Furthermore, zinc is known to stabilize a complex between the metal-regulatory factor MTF1 and its DNA-binding site [50] and is discussed as taking part in metal sensing mechanisms reviewed by Waldron et al. [51]. The function as part of a metal sensing and regulatory mechanism of zinc is reasonable. Zinc is redox inactive and therefore the level of free zinc can be altered without harming cell components. We determined the release of zinc under exposure to manganese and iron, resulting in relatively higher inorganic zinc fractions. In our opinion, this is the result of competitive reactions for binding spots of various peptides, where zinc is replaced by divalent iron or manganese. Zinc-specific regulatory mechanisms for maintaining the metal homeostasis of different metals will be induced simultaneously to zinc excretory mechanisms. Therefore, zinc deficiency can be an indicator of an imbalance of metal homeostasis of different metals, such as manganese or iron. Alternatively, altered expressions or activity in metal transporters could explain the redistribution of metals. As discussed earlier, we see a decrease in total zinc after manganese and iron treatment, which indicates an activation of export transporters. However, total manganese and iron levels have not decreased under manganese or iron treatment, respectively. Alteration of transporter activity alone would not explain the alteration of the distribution between HMM, LMM, and inorganic phases, yet it may contribute to the maintenance of the metal equilibrium. Another possible explanation for shifts in the distribution after exposition to the metals zinc, iron, and manganese is the activation of stress response pathways. Certain proteins are demonstrated to be regulated by metal ions and oxidant stress, such as metallothionein over the transcription factor 1 (MTF-1) [52]. In general, it is likely that stress response pathways will influence the metal distribution between HMM, LMM, and inorganic species. However, this response pathway includes several steps from transcriptional activation, translation, and accumulation to protein expression. The whole process may last several hours and therefore is most likely not the explanation for the findings in acute treatment after half an hour of exposure time [53]. But studies on the kinetics of stress response pathways, especially in *C. elegans*, are incomplete; therefore, we cannot exclude this possible explanation. Conclusively, we need to consider that our findings may be the result of transporter regulation, stress response pathways, competition for binding sites of the proteins, or a mix of all three contributing to the changes in distribution. In our opinion, the interplay between different mechanisms such as the release of zinc from HMM and LMM after iron or manganese intake, followed by the activation of stress response pathways or transporter regulation is contributing to the sustaining of the metal equilibrium. Our experiments support this, but further investigation into this matter is needed.

#### Chronic treatment

**Fig. 4:**
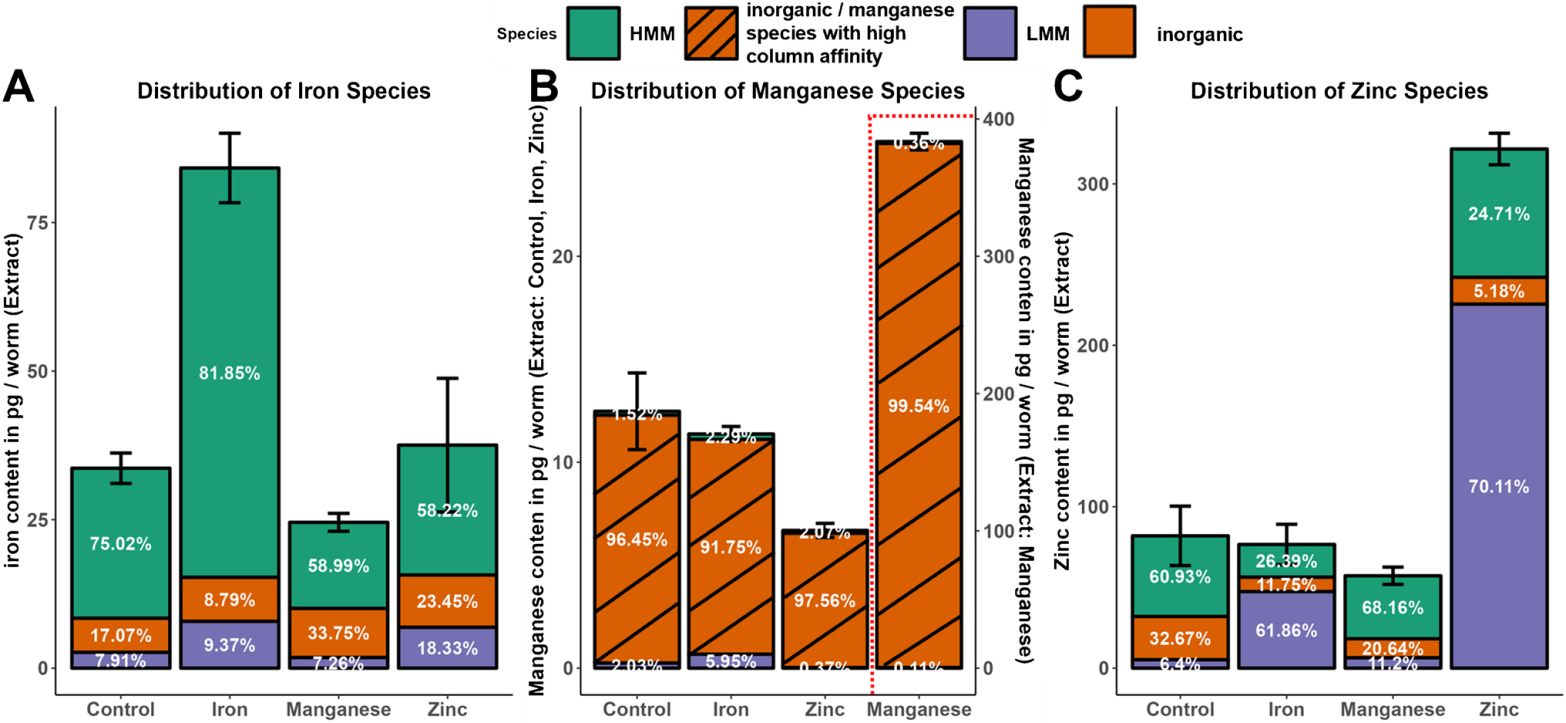
Distribution of high-molecular-mass (HMM), low-molecular-mass (LMM), and inorganic Fe (**A**), Mn (**B**), and Zn (**C**) species after chronic Fe, Mn, Zn treatment, and control (*x*-axis). Determination of HMM, LMM, and inorganic species was carried out with SEC-ICP-MS and presented in % and against the total metal content normalized on the number of worms (*y*-axis) determined in the extract with ICP-OES.

In YA the distribution of iron species is like in L1. The distribution of LMMs is slightly decreased. The distribution of HMMs has increased. Iron exposure leads to a significant elevated iron-level in the extract. Remarkably, the iron content in the inorganic fractions is relatively stable, whereas the iron bound to HMMs increases (Fig. 4A). This is reasoned in the high storage capacity of ferritin in the HMM fraction [54]. Manganese and zinc exposure decreases the HMM-bound iron. Zinc exposure increases the inorganic iron distribution and the LMM distribution, whereas manganese exposure increases the distribution of inorganic iron (Fig. 4B). In YA the dominant manganese species is inorganic or loosely bound. It complexes with ligands, such as citrate [55, 56] inside of the cytosol and cell organelles. As discussed, we noticed a shift for inorganic manganese species to higher retention times. From the experiments we concluded that manganese might be bound to hydrophilic proteins localized in the inside membrane of the Golgi apparatus. Thus, proteins would bind to the column material and slowly release manganese under the experimental conditions. Because of the technical limitations we defined the content of this fraction as inorganic manganese and/or loosely bound manganese to a species with high affination to our column material under the given elution conditions. We name it in the following labile manganese pool. Exposure to manganese increases the level of manganese in the extract significantly. The absorbed manganese is mainly added to the labile manganese pool. This agrees with our prior findings determined with an orthologue hyphenated instrument setup [23]. Interestingly, exposure to iron increases the manganese bound to LMMs slightly (Fig. 4B). In YA more than half of zinc is bound to HMMs, a third are inorganic and below 10% of zinc is bound in LMMs. This pattern changes if treated with iron or zinc to more than half of zinc bound to LMMs, respectively. We assume that this pattern is caused by MT expression (Fig. 4C), because MT expression is induced by heavy metal exposure, such as cadmium, copper, lead and nickel presented by Chen et al. [57]. MTs are with 0.5 to 15 kDa defined as LMMs. Their function is binding heavy metals to prevent cellular damage and deactivating oxidative agents. However, iron is not revealed to increase MT expression, our results indicate that MTs-like proteins or LMMs, capable of binding zinc and iron are expressed under iron exposure.

### Metabolomic studies with FT-ICR-MS

**Fig. 5:**
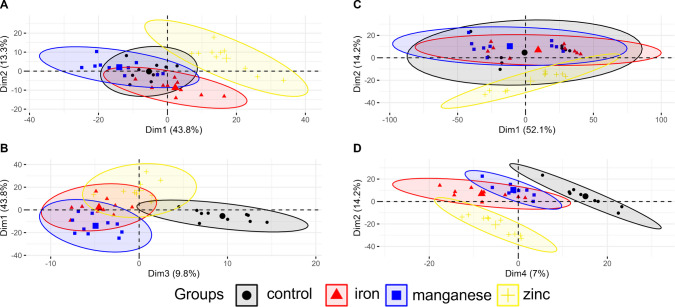
PCA diagram of chronically treated worms along dimensions 1 and 2 (**A**) and along dimensions 1 and 3 (**B**). PCA diagram of acutely treated L1 along dimensions 1 and 2 (**C**) and along dimensions 2 and 4 (**D**)

Metabolomics was followed with mass spectrometry using FT-ICR-MS and data preprocessing, resulting in 2920 m/z features converted to 2740 elemental compositions on chronic treatment and 10,200 m/z features converted to 5940 elemental compositions after acute treatment, respectively. The elementary composition was conducted in the CHNOSP space. The discrepancy in observed features between young L1 nematode (acute treatment) and young adults (chronic treatment) was expected, since the adult nematodes’ extract is very rich in lipid species, suppressing other signals when using direct injection techniques. The data after acute and chronic treatment were merged into separate matrices for multivariate statistics. Principal component analysis (PCA) was conducted over all annotations, after excluding annotations containing NA values in one or more samples (*n* = 413 for chronic and *n* = 1498 for acute treatment), which showed distinct separation for chronic treated worms along dimensions 2 and 1 (Fig. 5A), particularly evident between control and zinc-treated worms. This observation is consistent with our previous findings, indicating a notable disparity in the development of worms subjected to zinc treatment. This disparity is attributed to the avoidance behavior exhibited by the worms towards a food source with elevated zinc concentration. Interestingly, the PCA indicates discernible differences for manganese and iron-treated worms compared to the control in dimensions 1 and 3 (Fig. 5B). For acutely treated worms, the PCA does not reveal a distinct separation between the different treatment conditions in dimensions 1 and 2. In conclusion, the observed samples have a substantial overall degree of similarity in their metabolomic profiles (Fig. 5C). This is since the treated L1 worms are freshly hatched from the same pool and were exposed to experimental conditions for a very brief duration. Interestingly, the PCA reveals distinct separation for the different treatment concentrations in dimensions 2 and 4 (Fig. 5D). This could be due to the exposure to different metals and immediate alteration of the metabolomic composition of the organism.

**Fig. 6:**
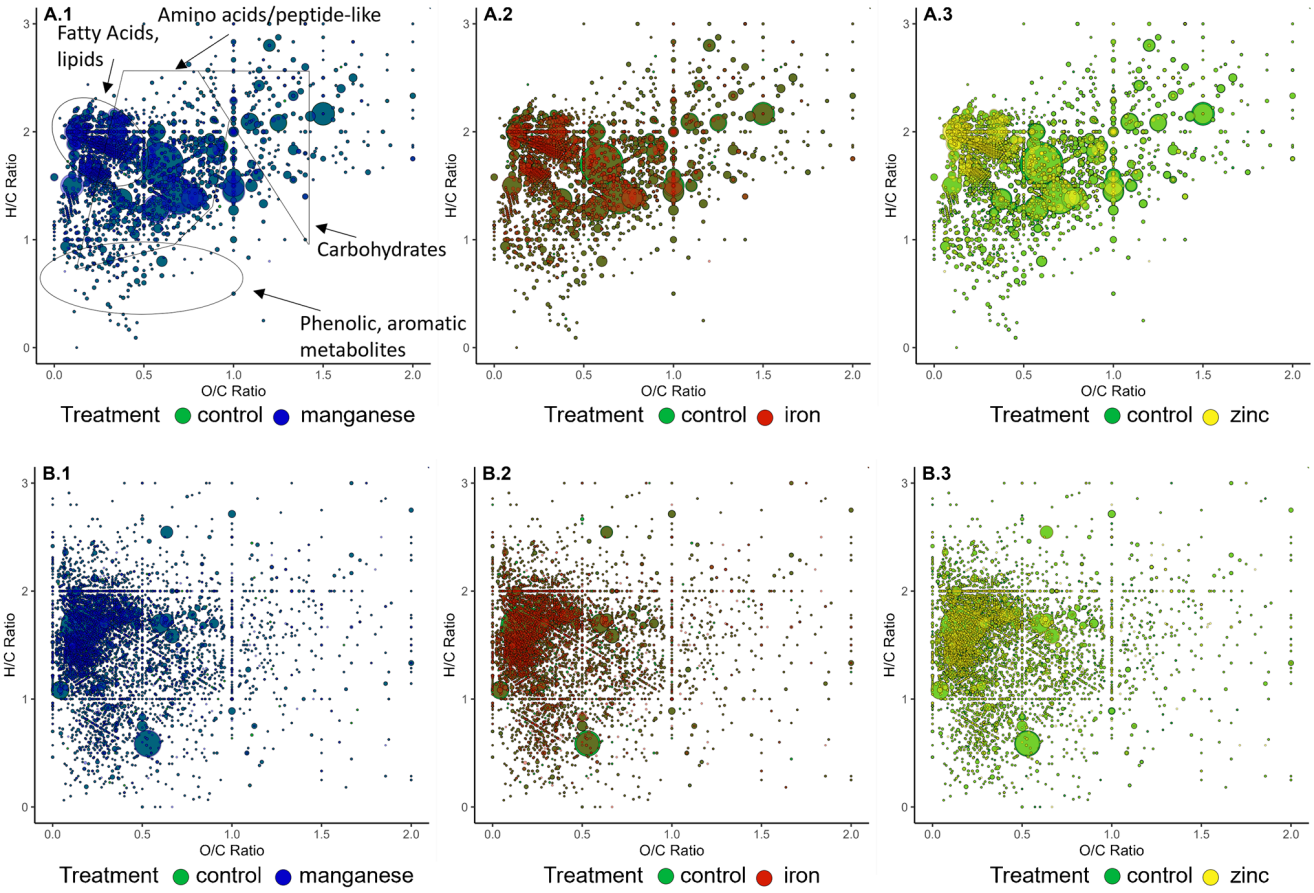
Van Krevelen diagram of about 3000 annotations for chronic treatment experiments (**A.1–3**) and about 6000 annotations for acute treatment experiments (**B.1–3**). Presented are areas for molecule groups such as fatty acids, lipids, amino acids-/peptide-like, carbohydrates, phenolic-/aromatic-like metabolites based on the ratio of H/C and O/C (A.1, exemplary). The different colors represent the group of samples: Control (green), manganese-treated (blue **A.1**, **B.1**), iron-treated (red **A.2**, **B.2**), or zinc-treated (yellow **A.3**, **B.3**) *C. elegans*. The size is correlated with the intensity determined by FT-ICR-MS in a range of 0.5 to 15-point size

The Van Krevelen diagram is a powerful approach to visualize differences in the chemical composition of samples with high complexity, such as extracts from biological systems. About 3000 molecular formulas for chronic treatment (Fig. 6A) and about 6000 molecular formulas for acute treatment (Fig. 6B) are visualized. The formulas were obtained from spectral data after they were processed, cleaned, and annotated using network-based annotation methods. Fatty acids, lipids, and amino acids, peptide-like molecules dominate in number of annotated molecules. Visualization confirms the results from the PCA for chronic and acute treatment, with overall great similarity of the different sample groups, only with the zinc-treated samples differing from the other groups. However, the samples of worms exposed to iron and manganese display certain molecules that diverge from those in the other three groups. Those molecules could potentially indicate metal-specific biomarkers for metabolic changes in response to chronic treatment. Overall, the treatment experiments show good similarity in the visual representation as a Van Krevelen diagram, allowing the comparisons of treated samples and control with further statistical approaches.

**Fig. 7:**
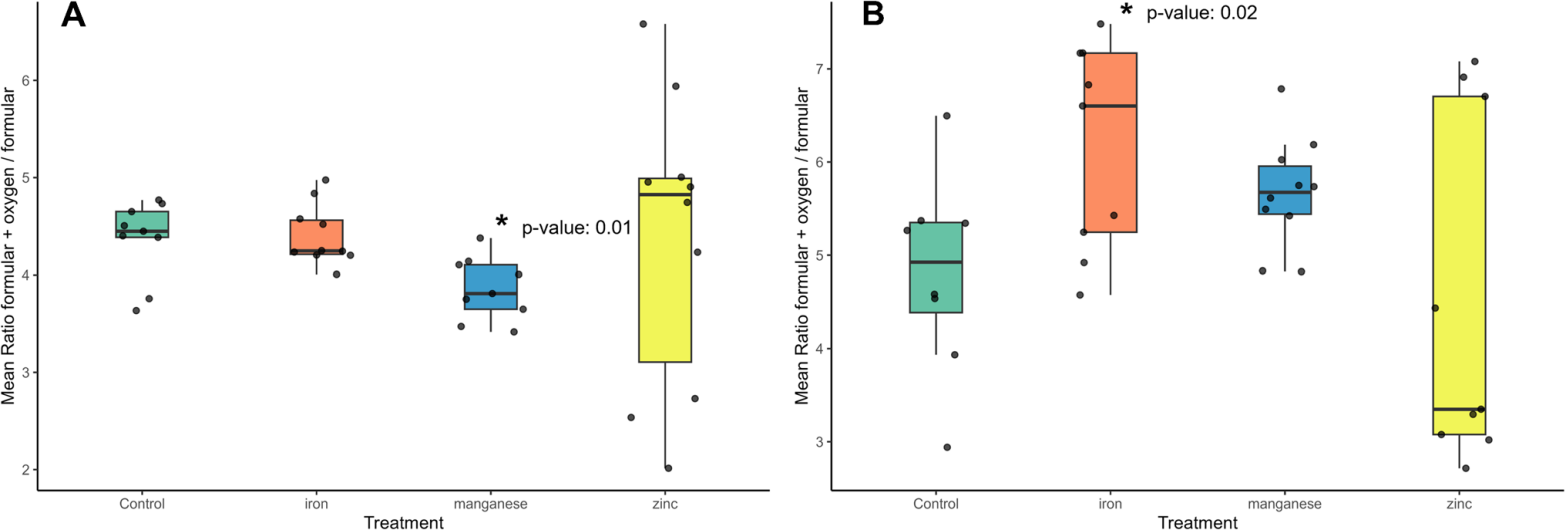
Mean value intensity of annotations (molecular formular + O) divided by the intensity of molecular formular as a general marker for unspecific oxidative processes after acute treatment is graphically represented as a boxplot (*n* = 10). The Grubbs outlier test was performed once, a Student’s *t*-test was performed while considering *p*-values < 0.05 as statistically significant. A significant decrease in this ratio was observed for chronically manganese treated worms (**A**, *p*-value 0.01), and significant elevation was observed for acutely iron treated worms (**B**, *p*-value 0.02)

Following the hypothesis that exposure to manganese or iron might lead to metabolic changes based on oxidative stress, we used the exact mass difference of one oxygen atom to determine possible oxidation products by investigating the annotations [formular + oxygen] in our data sets. By dividing the intensities of the [formular + oxygen] by the formular intensity, we got ratios indicating the insertion of oxygen in the molecule. The mean value of these ratios for each set of exposure experiments is presented in Fig. 7. Higher ratios would indicate oxidative processes in our experimental setup likely induced by oxidative stress throughout metal exposure. For chronic manganese treatment, a significant decrease in this value was observed (Fig. 7A). For this finding, we do not have any biological explanation since we expected higher values. Although we observed lower LMM iron concentration for manganese-treated worms in the chronic treatment, which might be an explanation for this finding. For acute treatment, a significant increase of the ratio towards [Formular + Oxygen] is observed for iron-treated worms and a slight increase for the manganese treatment, whereas zinc does not show any alteration compared with the control. These findings could be due to iron or manganese-induced oxidation (Fig. 7B). It agrees with our prior findings: unbound or loosely bound iron, determined with speciation analysis.

**Fig. 8:**
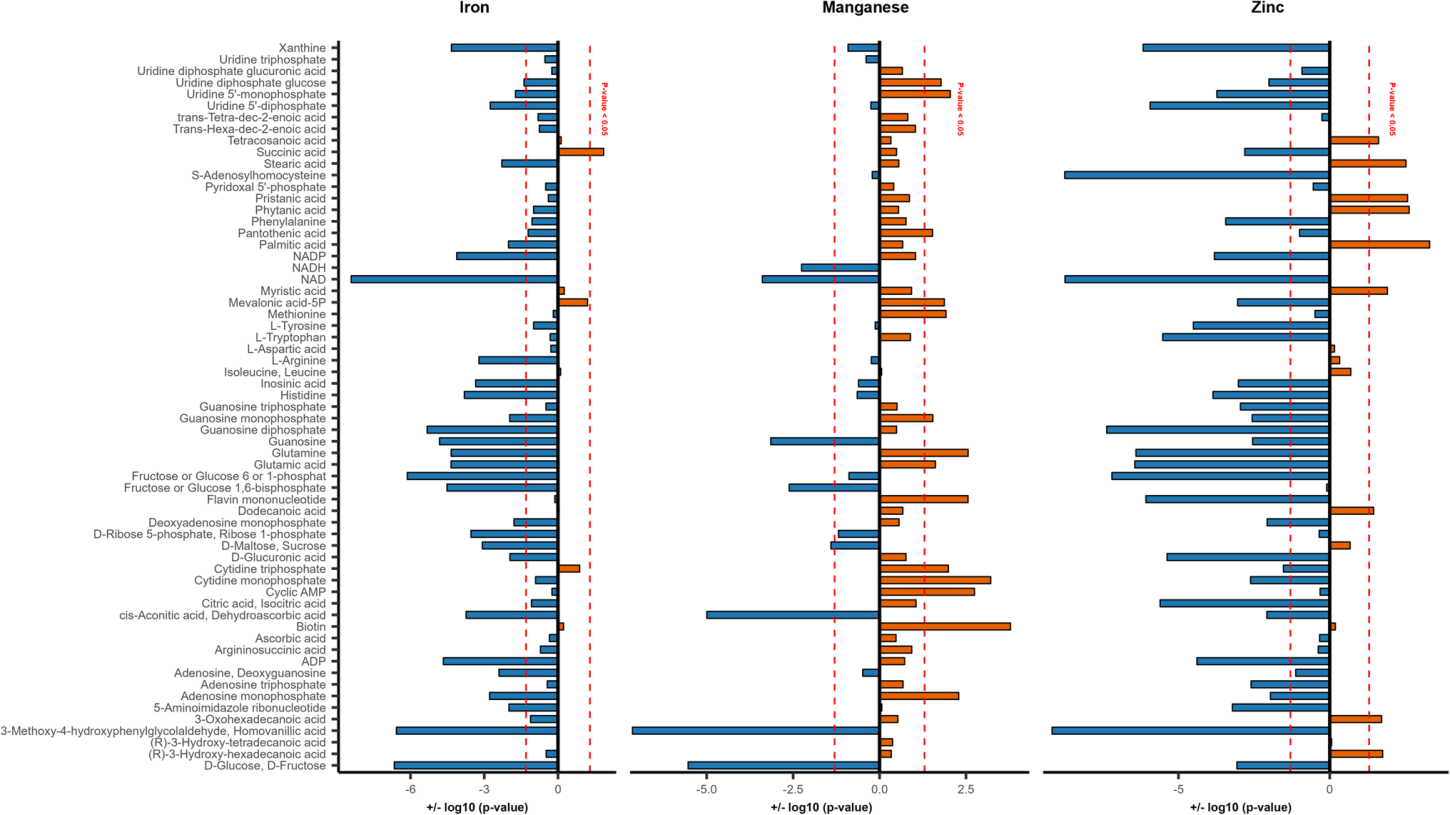
Differences between the intensities of biologically active molecules after exposure compared to the control group. The Student’s *t*-test was performed to compare the intensities of all the treatment groups to the control. The logarithm of the *p*-values (log *p*-values) was calculated for better graphical representation. For features with lower intensities, the values were multiplied by −1 to enhance visual contrast. In the resulting plot, lower intensities are represented in blue, while higher intensities are shown in orange. A red dashed line indicates the threshold for statistical significance (*p*-value = 0.05), corresponding to both the positive and negative log-transformed *p*-value. Bars crossing this threshold represent molecules whose intensities differ significantly from those of the control group

Given the biological origin of the samples, we used the HMDB database to match molecular formula annotations with actual compounds that are known to be part of biochemical pathways. A Student’s *t*-test was carried out to compare each treatment group (manganese, iron, and zinc) with the control. An overview is given for the chronic exposure, where defined molecules are compared to the control (Fig. 8). Interestingly, 57 or 50 out of 68 of the presented compounds show a lower intensity compared to control for iron- and zinc-treated worms, respectively, whereas, for manganese-treated worms only 23 out of 68 compounds had lower intensities compared to the control. This indicates different effects of manganese and iron, both oxidative active metals, on the organism. For iron-treated organisms, the precursors for Acetyl-CoA from the main synthesis pathways: glycolysis (Glucose), beta oxidation (stearic acid), protein degeneration (Leucine) are exhausted, whereas succinic acid levels are elevated. This indicates a high energy demand in the form of GTP consumption because of excessive iron intake. The transformation of succinic acid to fumarate seems to be here the bottleneck of the tricarboxylic acid cycle (TCA) cycle, and therefore it is accumulated (*p*-value 0.01, BH-adjusted 0.03) under chronic excessive iron intake. Accumulated succinic acid generally indicates an overload of the TCA cycle, an inhibition of succinate dehydrogenase (SDH), or both. Metabolite intensities for D-Ribose 5-phosphate involved in the pentose phosphate pathway (PPP) are significantly lower (*p*-value: 0.0003, BH-adjusted *q*-value: 0.0001) in iron-treated worms. The PPP is an important cellular metabolism while providing precursors for amino acid and nucleotide biosynthesis or balancing the redox equilibrium by providing reducing molecules [58]. An exhaustion or inhibition of the PPP is linked directly to increased cellular damage induced most likely by oxidative stress. Since we do not provide the determination of oxidative stress markers in this study, the direct link between our findings and oxidative stress remains hypothetical. However, different studies have shown or associated increased Mn or Fe levels with increased ROS in *C. elegans*, respectively [59–61].

Mechanisms for repairing or preventing oxidative stress induced cellular damage are as follows: DNA repair, protein repair or degradation, protection of biological membranes, antioxidative systems. Antioxidants such as uric acid, glutathione, ceruloplasmin, and ascorbic acid prevent oxidative damage in the aqueous phase of the cells. The precise biosynthesis pathway for ascorbate synthesis in *C. elegans* is not yet discovered, but recent findings with C_13_ labelling prove the capability of *C. elegans* to synthesize ascorbate within the organism [62] most likely under GTP consumption in an alternative pathway. Base Excision Repair uses ATP for the activation of DNA ligases in long-patch repair [63]; nucleotide excision repair hydrolyzes ATP to form a stable preincision form of the DNA-protein complex [64]; mismatch repair consumes ATP for damage recognition and repair by enzymes like MutS [65]. For protein repair or degradation, ATP is either directly used or indirectly over NADPH via the thioredoxin system [66]. The biosynthesis and repair of membrane lipids are complex and ATP-demanding mechanisms reviewed by E. Cronan, Jr. et al. [67]. Altogether, it is fair to say that the metabolic reaction to oxidative stress is highly energy demanding, supporting our findings after chronic iron exposure. Neurons have higher energy demands under normal conditions due to their need to maintain ion gradients for action potential propagation, support synaptic transmission by releasing and up-taking neurotransmitters such as dopamine, or just sustaining complex cell structures. Therefore, they are particularly vulnerable to iron-induced oxidative stress, as the mechanisms for preventing or repairing oxidative damage further exhaust the energy resources of the organism.

## Conclusion

This study indicates the alteration of metal homeostasis after induced imbalance by treatment with specific metals such as zinc, manganese, and iron. The data suggests that zinc plays a key role in maintaining the balance, particularly by regulating the levels of inorganic manganese and iron. Our findings further suggest that manganese and iron may compete with zinc for binding sites on high- and low-molecular mass (HMM and LMM) ligands. By providing binding sites of the HMM and LMM for redox-active manganese or iron, zinc ions would be released into the inorganic fraction, potentially reducing oxidative damage. Furthermore, elevated inorganic zinc levels following manganese intake appear to trigger zinc excretion and likely activate additional metal-regulatory mechanisms as a protective measure against oxidative stress. An interplay between stress response pathways and transporter activation may also be part of the explanation for our findings. Metabolomic analysis using FT-ICR mass spectrometry revealed shifts in biologically active molecules which, in the case of iron exposure, indicate an increased energy demand in the form of guanosine triphosphate (GTP). Elevated levels of succinic acid indicate a bottleneck in the biological pathway in the case of energy depletion after iron exposure. This increased energy requirement may reflect cellular efforts to prevent or repair damage induced by iron toxicity. If these processes fail or are exhausted, they could lead to downstream events such as cellular dysfunction, ferroptosis, or apoptosis.

Neurons, which are high energy-demanding cells, may be especially vulnerable to energy exhaustion induced by an imbalance of iron homeostasis. Therefore, neurodegenerative disorders, such as Parkinson’s disease, may originate, in part, from imbalances in metal homeostasis. However, further investigation into both the causes and consequences of imbalanced metal homeostasis is warranted. Results obtained with *C. elegans* wild type should be interpreted cautiously, as they cannot be directly extrapolated to human disease progression. Additional studies using different *C. elegans* strains, such as α-synuclein-expressing strains, as well as other animal models, are important to strengthen the possible link between metal imbalance and PD progression presented in this study.

## Supplementary Information

Below is the link to the electronic supplementary material.

Supplementary Material 1 (DOCX 356 KB)

Supplementary Material 2 (XLSX 4.02 MB)

Supplementary Material 3 (XLSX 9.20 MB)

## Data Availability

The data supporting the findings of this study are available within the manuscript and its supplementary materials. Additional data that support the results of this study are available from the corresponding author upon reasonable request.
