# Supplementary material for: Combined metallomics and metabolomics reveal impact of metal homeostasis on biological pathways in C. elegans
Source: Anal Bioanal Chem. 2026 Jan 9;418(5):1471–85. doi: 10.1007/s00216-025-06306-z (PMC12909348; doi:10.1007/s00216-025-06306-z)
Supplement: Supplementary file 1 — Supplementary Material 1 (DOCX 356 KB) [file 216_2025_6306_MOESM1_ESM.docx]

# Supplementary Information


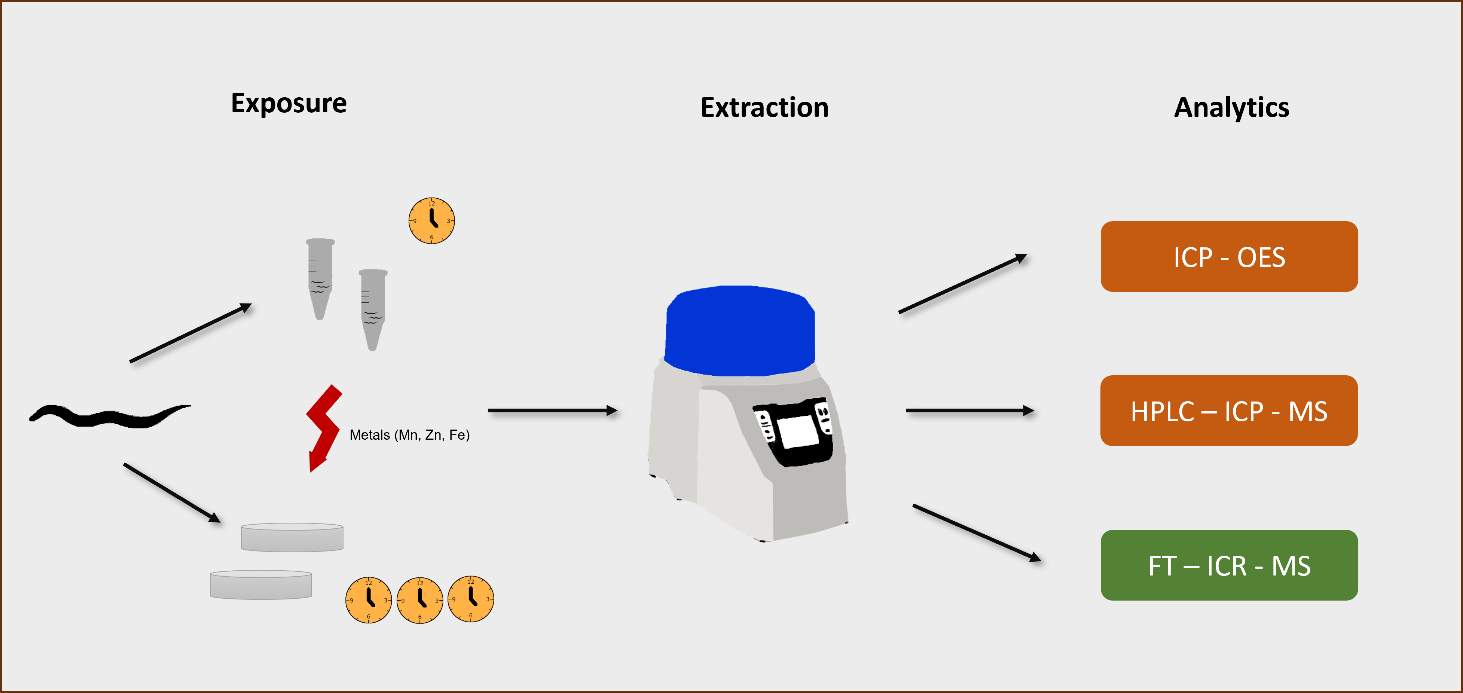


**Figure SI 1: Graphical presentation of experiments carried out in this study.**


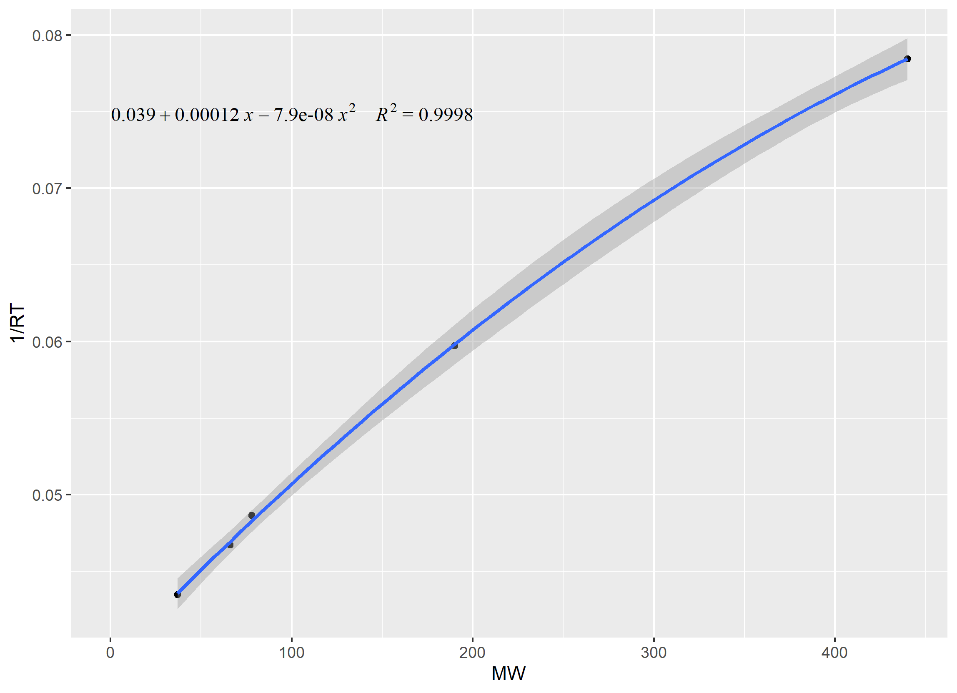


**Figure SI 2: Mass calibration for SEC – Chromatography for the chronical treatment experiments.**

Table SI 1: Data for mass calibration curve for chronical treatment experiments

| Species | MW (kDa) | RT (min) |
| --- | --- | --- |
| Ferritin | 440 | 12,747 |
| y-globulin | 190 | 16,743 |
| transferrin | 78 | 20,551 |
| HSA | 66 | 21,403 |
| B-lactoglobulin | 37 | 23,005 |


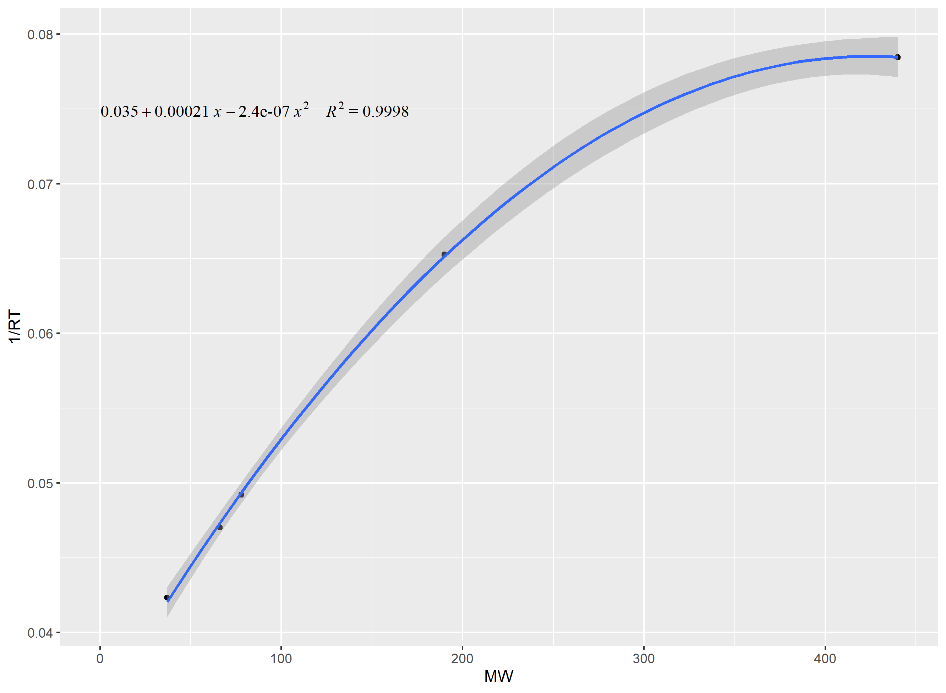


Figure SI 3: Mass calibration for SEC – Chromatography for the acute treatment experiments.

Table SI 2: Data for mass calibration curve for active treatment experiments

| Species | MW (kDa) | RT (min) |
| --- | --- | --- |
| Ferritin | 440 | 12,747 |
| y-globulin | 190 | 15,3185 |
| transferrin | 78 | 20,3155 |
| HSA | 66 | 21,2645 |
| B-lactoglobulin | 37 | 23,61633 |

**SI 1:** R-Script for reprocessing FT-ICR-MS data using the in-house R-package.

##neg - Mode

library(bgcDH)

library(tidyverse)

library(magrittr)

wd <- dirname(rstudioapi::getSourceEditorContext()$path) %>%

setwd()

setwd(paste0(wd,"/export/neg/20221028_Without md"))

##check headers!! check error scales according to dataset!

wiggles_bk(input.file = ".ascii", header = TRUE, sensitivity = 3.5)

iso_filter(input.file = "last", header = TRUE, error = 0.2, iso = c("13C", "34S", "37Cl"))

mdfilter_2(input.file = "last", error = 0.6, header = TRUE, adducts = c("[M-2H]","[M-H]-", "[M+Cl]-"))

align_peaks(input.file = "last", header = TRUE, ppm.win = 0.5)

M = read.table(paste0(wd, "/export/neg/wiggles_bk-filtered/iso_filter-filtered/md_filter-filtered/Matrix.txt"), header = T)

colnames(M) = stringr::str_remove(colnames(M), ".ascii")

setwd(wd)

openxlsx::write.xlsx(M, "2022-10-28-Matrix-Wurms-neg.xlsx")

**SI 2:** Mass data obtained from FT-ICR-MS experiments in .xlsx files for chronical (SI 2.1) and acute treatment (SI2.2) experiments.

Table SI 3: p-values of metabolites of interest compared to control and q-values obtained with FDR correction (Benjamin Hochberg method)

| compound | p-values | | | q-Values (BH-Test) | | |
| --- | --- | --- | --- | --- | --- | --- |
|  | iron | manganese | zinc | iron | manganese | zinc |
| Succinic acid | 1,39E-02 | 3,22E-01 | 1,56E-03 | 3,09E-02 | 4,11E-01 | 3,65E-03 |
| Isoleucine, Leucine | 7,78E-01 | 8,73E-01 | 2,02E-01 | 7,91E-01 | 8,87E-01 | 2,62E-01 |
| L-Aspartic acid | 5,14E-01 | 9,81E-01 | 6,99E-01 | 6,04E-01 | 9,81E-01 | 7,36E-01 |
| Glutamine | 4,49E-05 | 2,74E-03 | 3,93E-07 | 2,52E-04 | 1,58E-02 | 3,43E-06 |
| Glutamic acid | 4,45E-05 | 2,42E-02 | 3,60E-07 | 2,52E-04 | 8,03E-02 | 3,43E-06 |
| Methionine | 6,44E-01 | 1,19E-02 | 3,25E-01 | 6,90E-01 | 4,70E-02 | 3,97E-01 |
| Histidine | 1,55E-04 | 2,25E-01 | 1,38E-04 | 7,18E-04 | 3,46E-01 | 5,27E-04 |
| Phenylalanine | 8,78E-02 | 1,71E-01 | 3,64E-04 | 1,50E-01 | 3,10E-01 | 1,17E-03 |
| cis-Aconitic acid, Dehydroascorbic acid | 1,83E-04 | 1,01E-05 | 8,33E-03 | 7,83E-04 | 2,12E-04 | 1,48E-02 |
| L-Arginine | 6,09E-04 | 5,79E-01 | 4,71E-01 | 2,15E-03 | 6,29E-01 | 5,31E-01 |
| Ascorbic acid | 4,49E-01 | 3,34E-01 | 4,61E-01 | 5,50E-01 | 4,12E-01 | 5,30E-01 |
| D-Glucose, D-Fructose | 2,19E-07 | 2,90E-06 | 8,52E-04 | 5,34E-06 | 9,13E-05 | 2,36E-03 |
| L-Tyrosine | 1,02E-01 | 7,48E-01 | 3,10E-05 | 1,65E-01 | 7,85E-01 | 1,35E-04 |
| 3-Methoxy-4-hydroxyphenylglycolaldehyde, Homovanillic acid | 2,67E-07 | 7,15E-08 | 6,65E-10 | 5,34E-06 | 4,50E-06 | 3,62E-08 |
| Citric acid, Isocitric acid | 8,34E-02 | 8,80E-02 | 2,48E-06 | 1,47E-01 | 2,22E-01 | 1,37E-05 |
| D-Glucuronic acid | 1,11E-02 | 1,72E-01 | 4,20E-06 | 2,56E-02 | 3,10E-01 | 1,97E-05 |
| Dodecanoic acid | 8,99E-01 | 2,12E-01 | 3,56E-02 | 8,99E-01 | 3,45E-01 | 5,05E-02 |
| L-Tryptophan | 4,82E-01 | 1,28E-01 | 3,02E-06 | 5,78E-01 | 2,65E-01 | 1,53E-05 |
| Pantothenic acid | 6,09E-02 | 2,93E-02 | 1,00E-01 | 1,18E-01 | 8,79E-02 | 1,36E-01 |
| trans-Tetra-dec-2-enoic acid | 1,55E-01 | 1,53E-01 | 5,52E-01 | 2,32E-01 | 2,91E-01 | 6,01E-01 |
| Mevalonic acid-5P | 6,27E-02 | 1,33E-02 | 9,01E-04 | 1,18E-01 | 4,95E-02 | 2,39E-03 |
| Myristic acid | 5,56E-01 | 1,18E-01 | 1,25E-02 | 6,33E-01 | 2,65E-01 | 2,00E-02 |
| Biotin | 5,91E-01 | 1,63E-04 | 6,51E-01 | 6,45E-01 | 2,58E-03 | 6,97E-01 |
| (R)-3-Hydroxy-tetradecanoic acid | NA | 4,20E-01 | 8,70E-01 | NA | 4,90E-01 | 8,84E-01 |
| Pyridoxal 5'-phosphate | 3,16E-01 | 3,89E-01 | 2,84E-01 | 4,31E-01 | 4,72E-01 | 3,53E-01 |
| Trans-Hexa-dec-2-enoic acid | 1,76E-01 | 9,17E-02 | 9,49E-01 | 2,57E-01 | 2,22E-01 | 9,49E-01 |
| Palmitic acid | 9,66E-03 | 2,14E-01 | 5,00E-04 | 2,52E-02 | 3,45E-01 | 1,53E-03 |
| Fructose or Glucose 6 or 1-phosphat | 7,41E-07 | 1,31E-01 | 6,32E-08 | 1,11E-05 | 2,65E-01 | 7,71E-07 |
| 3-Oxohexadecanoic acid | 7,62E-02 | 2,96E-01 | 1,94E-02 | 1,39E-01 | 4,05E-01 | 2,96E-02 |
| (R)-3-Hydroxy-hexadecanoic acid | 3,31E-01 | 4,59E-01 | 1,78E-02 | 4,32E-01 | 5,25E-01 | 2,78E-02 |
| Stearic acid | 5,21E-03 | 2,77E-01 | 3,03E-03 | 1,42E-02 | 3,95E-01 | 5,60E-03 |
| Argininosuccinic acid | 1,93E-01 | 1,17E-01 | 4,14E-01 | 2,76E-01 | 2,65E-01 | 4,95E-01 |
| Pristanic acid | 4,05E-01 | 1,37E-01 | 2,68E-03 | 5,06E-01 | 2,69E-01 | 5,36E-03 |
| Phytanic acid | 1,02E-01 | 2,82E-01 | 2,38E-03 | 1,65E-01 | 3,95E-01 | 5,27E-03 |
| Cytidine monophosphate | 1,23E-01 | 6,09E-04 | 2,42E-03 | 1,94E-01 | 6,40E-03 | 5,27E-03 |
| Uridine 5'-monophosphate | 1,87E-02 | 9,00E-03 | 1,86E-04 | 3,86E-02 | 4,05E-02 | 6,30E-04 |
| Fructose or Glucose 1,6-bisphosphate | 3,06E-05 | 2,43E-03 | 7,89E-01 | 2,29E-04 | 1,58E-02 | 8,16E-01 |
| D-Maltose, Sucrose | 8,36E-04 | 3,93E-02 | 2,12E-01 | 2,79E-03 | 1,13E-01 | 2,69E-01 |
| Adenosine monophosphate | 1,65E-03 | 5,11E-03 | 1,10E-02 | 5,23E-03 | 2,68E-02 | 1,81E-02 |
| Guanosine monophosphate | 1,09E-02 | 2,88E-02 | 2,72E-03 | 2,56E-02 | 8,79E-02 | 5,36E-03 |
| Tetracosanoic acid | 7,40E-01 | 4,69E-01 | 2,44E-02 | 7,72E-01 | 5,28E-01 | 3,63E-02 |
| S-Adenosylhomocysteine | NA | 6,14E-01 | 1,75E-09 | NA | 6,55E-01 | 3,62E-08 |
| Uridine 5'-diphosphate | 1,75E-03 | 5,61E-01 | 1,16E-06 | 5,25E-03 | 6,20E-01 | 7,07E-06 |
| ADP | 2,17E-05 | 1,88E-01 | 4,08E-05 | 1,86E-04 | 3,30E-01 | 1,66E-04 |
| Guanosine diphosphate | 4,76E-06 | 3,21E-01 | 4,25E-08 | 5,72E-05 | 4,11E-01 | 6,48E-07 |
| Flavin mononucleotide | 7,46E-01 | 2,76E-03 | 8,39E-07 | 7,72E-01 | 1,58E-02 | 5,69E-06 |
| Cytidine triphosphate | 1,31E-01 | 1,02E-02 | 2,93E-02 | 2,02E-01 | 4,28E-02 | 4,26E-02 |
| Uridine triphosphate | 2,96E-01 | 4,09E-01 | NA | 4,13E-01 | 4,86E-01 | NA |
| Adenosine triphosphate | 3,65E-01 | 2,09E-01 | 2,51E-03 | 4,66E-01 | 3,45E-01 | 5,27E-03 |
| Guanosine triphosphate | 3,24E-01 | 3,12E-01 | 1,11E-03 | 4,32E-01 | 4,11E-01 | 2,72E-03 |
| Uridine diphosphate glucose | 4,16E-02 | 1,66E-02 | 9,75E-03 | 8,32E-02 | 5,81E-02 | 1,65E-02 |
| Uridine diphosphate glucuronic acid | 5,61E-01 | 2,19E-01 | 1,21E-01 | 6,33E-01 | 3,45E-01 | 1,60E-01 |
| NAD | 3,84E-09 | 4,07E-04 | 1,78E-09 | 2,31E-07 | 5,13E-03 | 3,62E-08 |
| NADH | NA | 5,56E-03 | NA | NA | 2,70E-02 | NA |
| NADP | 7,63E-05 | 9,12E-02 | 1,54E-04 | 3,82E-04 | 2,22E-01 | 5,54E-04 |
| D-Ribose 5-phosphate, Ribose 1-phosphate | 2,89E-04 | 6,49E-02 | 4,48E-01 | 1,15E-03 | 1,78E-01 | 5,25E-01 |
| Adenosine, Deoxyguanosine | 3,99E-03 | 3,26E-01 | 7,48E-02 | 1,14E-02 | 4,11E-01 | 1,04E-01 |
| Xanthine | 4,61E-05 | 1,23E-01 | 6,80E-07 | 2,52E-04 | 2,65E-01 | 5,18E-06 |
| Guanosine | 1,51E-05 | 7,14E-04 | 2,82E-03 | 1,51E-04 | 6,43E-03 | 5,37E-03 |
| 5-Aminoimidazole ribonucleotide | 1,01E-02 | 8,57E-01 | 6,06E-04 | 2,53E-02 | 8,85E-01 | 1,76E-03 |
| Cyclic AMP | 5,70E-01 | 1,79E-03 | 4,79E-01 | 6,33E-01 | 1,41E-02 | 5,31E-01 |
| Deoxyadenosine monophosphate | 1,62E-02 | 2,72E-01 | 8,52E-03 | 3,48E-02 | 3,95E-01 | 1,48E-02 |
| Inosinic acid | 4,48E-04 | 2,46E-01 | 9,50E-04 | 1,68E-03 | 3,69E-01 | 2,41E-03 |
